# Relationship between birth weight and overweight/obesity among students in Florianópolis, Santa Catarina, Brazil: a retrospective cohort study 

**DOI:** 10.1590/1516-3180.2014.1325630

**Published:** 2014-07-22

**Authors:** Camila Elizandra Rossi, Francisco de Assis Guedes de Vasconcelos

**Affiliations:** I MSc. Assistant Professor, Undergraduate Nutrition Course, Universidade Federal da Fronteira Sul (UFFS), Chapecó, Santa Catarina, Brazil; II PhD. Titular Professor, Undergraduate Nutrition Course, Department of Public Health, Universidade Federal de Santa Catarina (UFSC), Florianópolis, Santa Catarina, Brazil

**Keywords:** Child, Adolescent, Birth weight, Overweight, Obesity, Criança, Adolescente, Peso ao nascer, Sobrepeso, Obesidade

## Abstract

**CONTEXT AND OBJECTIVE:**

Being born heavier than 4 kg is associated with current overweight and obesity over the long term. The objective here was to ascertain whether birth weight was related to overweight or obese status, among 7 to 14-year-old schoolchildren, taking into consideration the possible interactions between socioeconomic factors and other biological variables.

**DESIGN AND SETTING:**

Retrospective cohort study on a probabilistic sample of 2,696 children and adolescents living in Florianópolis, Santa Catarina, Brazil.

**METHODS:**

The following data were collected: anthropometric (student's weight, height and age; and parents' weight and height), socioeconomic (family income, number of people in house and parental schooling level), birth weight and gestational age. Overweight and obesity were classified using percentiles of body mass index and triceps and subscapular skinfolds. The outcome variables were overweight and obesity and the main explanatory variables were birth weight and birth weight according to gestational age. The control variables were the parents' nutritional status, their schooling level and the *per capita* family income. Poisson multivariate regressions were carried out.

**RESULTS:**

Higher prevalence of high birth weight was observed among overweight male adolescents (PR = 1.14; 95% CI = 1.02-1.27; P = 0.03), but this was not observed among obese male adolescents. Low birth weight and being born small for gestational age were also not associated with the outcomes. Among overweight and obese children, birth weight was not significantly different from that of normal-weight children.

**CONCLUSION:**

No significant association between birth weight and obesity was observed. However, there was a weak but significant association between high birth weight and overweight, among male adolescents.

## Abstract (pt)

**CONTEXTO E OBJETIVO:**

Nascer com peso > 4 kg se associa a sobrepeso e/ou obesidade em longo prazo. O objetivo foi verificar se o peso ao nascer foi um fator relacionado ao estado de sobrepeso ou obesidade atuais, em escolares de 7-14 anos, levando em consideração interações possíveis com fatores socioeconômicos e outras variáveis biológicas.

**TIPO DE ESTUDO E LOCAL:**

Estudo coorte retrospectivo numa amostra probabilística de 2.696 crianças e adolescentes residentes em Florianópolis, Santa Catarina, Brasil.

**MÉTODOS:**

Coletaram-se dados antropométricos (peso, estatura e idade dos escolares, peso e estatura dos pais), socioeconômicos (renda familiar, número de pessoas na casa, escolaridade dos pais) e de peso ao nascer e idade gestacional. Sobrepeso e obesidade foram definidos utilizando-se os percentis de índice de massa corporal e as dobras cutâneas tricipital e subescapular. Sobrepeso e obesidade foram desfechos, e peso ao nascer e peso ao nascer/por idade gestacional foram variáveis explanatórias principais. As variáveis de controle foram: estado nutricional dos pais, seu nível de escolaridade e renda familiar *per capita*. Foram realizadas regressões de Poisson.

**RESULTADOS:**

Nos adolescentes do sexo masculino com sobrepeso, houve maior prevalência de elevado peso ao nascer (RP = 1,14; 95% IC = 1,02-1,27; P = 0,03). Naqueles com obesidade, não foi observado o mesmo. Baixo peso ao nascer e nascer pequeno para a idade gestacional também não se associaram aos desfechos. Nas crianças com sobrepeso ou obesidade, o peso ao nascer não diferiu daquele entre as eutróficas.

**CONCLUSÃO:**

Não se observou associação significativa entre peso ao nascer e obesidade. Porém, houve associação significativa, ainda que fraca, entre elevado peso ao nascer e sobrepeso, nos adolescentes do sexo masculino.

## INTRODUCTION

Among the factors associated with obesity, significant attention has been given to birth weight. High birth weight (HBW), especially greater than or equal to 4 kg,^1^ has been correlated with overweight and/or obesity over the long term, in epidemiological studies.^2^ ^-^ ^5^ Particularly in developed countries, HBW is the main risk factor for obesity among children and adolescents whose mothers were affected by gestational diabetes mellitus, because of the elevated number of adipose cells acquired by the infant.^6^

However, other factors may overlap the association between birth weight and overweight/obesity, such as the mother's body mass index (BMI),^7^ both the mother's and the father's BMI,^8^ family income^3^ ^,^ ^8^ and the type of school attended, with regard to developing countries.^9^ These factors may reduce the importance of the birth weight variable in multivariate analysis.^3^ Systematic reviews have shown that correlations between birth weight and overweight/obesity among children not only have presented contradictory results, but also have differed regarding the methods used. Thus, these reviews have demonstrated that research on this subject needs to be conducted with the proper methodological rigor, in order to identify factors that might reduce the effect of birth weight on overweight or obesity among children and adolescents.^10^ ^,^ ^11^

Because of the contradictions in these studies, it is important to clarify the association between birth weight and overweight/obesity, so as to determine the stage of life at which this relationship first appears. Longitudinal studies have revealed these associations in adulthood.^12^ ^,^ ^13^

A study conducted in the city of Florianópolis in the year 2002 by de Assis et al.^14^, among children aged 7-9 years, found that the prevalence of overweight (including obesity) was 22.1%, in accordance with the criteria of Cole et al.^15^ Data from this city's live births registry, obtained in 2005, showed that the prevalence of low birth weight (LBW) among live births in Florianópolis was 8.3%.^16^ These proportions are similar to those found in developed countries, where an increase in HBW has been observed with a simultaneous decrease in LBW.^17^ ^,^ ^18^ Florianópolis was classified as the fourth highest city in Brazil in terms of human development in 2000 and the third city in 2013.^19^ ^,^ ^20^ Thus, the importance of the present study lies in the fact that in Florianópolis, elevated HBW prevalence that could be correlated with high rates of overweight and obesity would be expected.

## OBJECTIVE

The purpose of this study was to investigate whether overweight and obese children and adolescents aged 7 to 14 years living in Florianópolis, Santa Catarina, southern Brazil, were born with low or high birth weight, taking into consideration the possible interactions between socioeconomic factors and other biological variables.

## METHOD

### Design, setting and ethics

This study was conducted on a retrospective cohort from the year 2007. An assessment was made on a probabilistic sample of schoolchildren aged 7 to 14 years who were enrolled in public and private elementary schools in Florianópolis, Santa Catarina, southern Brazil. The schoolchildren in this investigation were included after obtaining consent from their parents or legal guardians, who signed a free and informed consent statement. This study had previously been approved by the Ethics Committee for Human Research of the Federal University of Santa Catarina (Universidade Federal de Santa Catarina, UFSC) through project number 028/06.

### Sample

The sampling was divided into two stages. Firstly, the 221 schools in Florianópolis were listed according to their geographical location in the city (north, south, east, center or mainland) and their kind of institution (public or private). The number of schools selected in each of the four geographical areas was defined taking into account: the proportion of schools in each area compared to the totality of schools in the city and the proportion of private and public institutions in each geographical area, totalizing 17 schools (11 public and 6 private). The selection of the 17 schools was made randomly, by simple draw, but taking into account the previous list (stratified selection by geographical area and kind of school). Subsequently, students in each school were selected taking into consideration the ratios of schoolchildren registered in the 2004 school census in Florianópolis (53,595 individuals) in the following categories: geographical location of the student's home, kind of school, gender and age group.

Sample size was calculated taking the prevalence of overweight (including obesity) among children aged 7 to 9.9 years to be 10%,^21^ and 17% for adolescents aged 10 to 14 years,^22^ with 95% confidence levels and a two-tailed sampling error of 2%. The design effect was estimated to be 1.3 and the power was taken to be 80%. This calculation resulted in a requirement for a sample size of 1,100 children aged 7 to 9.9 years and 700 adolescents aged 10 to 14.99 years. In addition, all the children who had participated in a previous study conducted in 2002,^14^ and who in 2007 were adolescents enrolled in these randomly selected schools were also included. It was possible to locate 30% of all the students who had participated in the previous study in these randomly selected schools.

Considering the error margins for losses in tests, the total sample was estimated to be 1,200 children and 1,900 adolescents (800 new adolescents plus 1,100 adolescents from the previous study). Data on 2,863 students were collected. Those younger than seven years of age (n = 18) and those who were 15 years or over (n = 16) were excluded, as were those for whom no valid data for weight (n = 2) or birth weight (n = 131) was available. Thus, the final sample investigated comprised 2,696 students (857 children and 1,839 adolescents), i.e. 94% of the total. 

### Data collection

Biological data (gender, age, birth weight, gestational age, weight, height, and subscapular and triceps skinfolds of the schoolchildren; and age, weight and height of their parents) and socioeconomic data (kind of school, i.e. public or private, family income level and parental educational level) were collected.

Information relating to birth weight, gestational age, age, parents' weight and height and socioeconomic data were collected by means of a self-administered questionnaire that was sent to the parents and legal guardians of the students.

Anthropometric data on the students were collected by previously trained anthropometry technicians,^23^ following a protocol based on the recommendations of Lohman.^24^ ^,^ ^25^ Weight was assessed using a Marte electronic scale, model PP 180 (Marte Científica, Santa Rita do Sapucaí - MG, Brazil). with a capacity for 180 kg and precision of 100 grams. Height was measured using an AlturExata stadiometer (AlturExata, Belo Horizonte - MG, Brazil) with 1.0 millimeter precision. Skinfolds were measured using a Cescorf caliper (Cescorf Equipamentos Antropométricos, Porto Alegre - RS, Brazil) with 0.1 millimeter precision. The children and adolescents were measured without shoes and wearing light clothes. 

A pilot study was conducted among subjects who were not included in the sample and, following the recommendations from the World Health Organization,^26^ the intra-rater technical measurement error (TME) for skinfold (SF) measurements was calculated. The intra-rater TME showed a reliability coefficient (R) greater than 0.95, which showed that all of the anthropometry technicians made skinfold measurements properly, thus resulting in low variability in the data.^23^

The data were entered into EpiData 3.2 and were fully checked by the duly trained data entry team, and automatic consistency and amplitude checks were made. 

### Statistical variables and analysis

The outcome variables were overweight and obesity. Overweight was defined as BMI ≥ 85th percentile, according to age and gender, as proposed by Must et al.27 and as recommended by the Brazilian Ministry of Health until the year 2008.28 Obesity was defined as BMI ≥ 85th percentile, according to age and gender, as proposed by Must et al.,27 along with triceps and subscapular skinfolds in millimeters (mm) ≥ 90th percentile as proposed by Johnson et al.29 The criteria of Must et al.27 were chosen because they are widely used in the literature and have been recommended by the World Health Organization.24 These were also recommended by the Brazilian Ministry of Health28 for evaluating nutritional status among children and adolescents until 2008, when this study was conducted.

Birth weight and birth weight according to gestational age were the independent variables (or exposure) and were classified as follows: a) birth weight was classified as a single piece of data, as LBW (< 3,000 g), appropriate birth weight (ABW; between 3,000 and 3,999 g) and HBW (≥ 4,000 g);^24^ ^,^ ^30^ and b) birth weight was correlated with gestational age in order to classify schoolchildren as small for gestational age (SGA), i.e. below the 10^th^ percentile; appropriate for gestational age (AGA), i.e. between the 10^th^ and the 90^th^ percentiles; and large for gestational age (LGA), i.e. above the 90^th ^percentile.^30^ The World Health Organization classifies children as presenting insufficient birth weight if they are born weighing between 1,500 and 2,999 g and as presenting low birth weight if they are born weighing 2,500 g or less.^24^ In our study, these categories were unified because the number of responses was low and this could have hidden an association between the outcome and the independent variables.

The control variables analyzed were the following: age and gender of the students; their parents' education level classified into three categories (< 8; 8 to 11; or ≥ 12 years of education), based on the recommendations of the Brazilian Association for Population Studies (Associação Brasileira de Estudos Populacionais, ABEP);^31^ per capita family income in quartiles (in Brazilian money); kind of school (public or private); and parental BMI. The socioeconomic variables of parental education, per capita income and kind of school, and the parental BMI, were considered to be control variables. 

The BMI of the students and their parents was obtained by dividing the weight measurement (in kg) by the square of their height (in meters). Non-elderly parents were classified as overweight if their BMI was between 25 and 30 kg/m^2^ or obese if their BMI was ≥ 30 kg/m^2^, as proposed by the World Health Organization,^24^ and elderly parents were classified as overweight if their BMI was ≥ 27 kg/m^2^, as proposed by the American Dietetic Association.^32^ Both of these sets of criteria are recommended by the Brazilian Ministry of Health.^28^

An analysis on the consistency of the database was made using the Stata version 9.0 statistical package. 

A descriptive analysis was conducted to show the prevalence ratios of overweight and obesity for each independent variable, and the prevalence of LBW, HBW, SGA and LGA.

In an inferential analysis, models divided by age group and gender were used to calculate associations among children aged 7 to 9.9 years and, separately, among adolescents 10 to 14.9 years, because of the possible effect of sexual maturation among the adolescents and the differences between genders regarding the prevalences of overweight and obesity. In order to compare the prevalences of overweight and obesity for the different categories within each variable studied, an analysis was performed using Pearson's modified contingency coefficient, based on chi-square statistics. 

Poisson univariate analysis was used to investigate association between birth weight (independent variable) and overweight and obesity (outcomes). Multivariate analysis was performed to ascertain the extent to which the exposure variables influenced the outcomes. Additionally, 95% confidence interval (95% CI) and P-values were estimated. The Poisson analysis model was used because for high-prevalence outcomes in cross-sectional studies (more than 10%), odds ratio estimates are said to either overestimate or underestimate associations with outcomes, in comparison with prevalence ratios.^33^ ^,^ ^34^

All the analysis took into consideration the effect of the sampling design, through the *svy *command in the Stata software, which is used to analyze data from complex samples. Associations among the variables for which the P-value was ≤ 0.05 were considered to be statistically significant. 

## RESULTS

Data on 2,863 children (aged 7-9 years) and adolescents (aged 10-14 years) were collected. The proportion of the data that comprised refusals or exclusions was 5.8%. Students less than 7 years of age (n = 18) and more than 14.9 years of age (n = 16) were excluded because these ages were outside of the study range. Invalid weight data (less than 10 kg, n = 2) and birth weight (lower than 800 grams, n = 131, and higher than 6 kg) were also excluded. Occurrences of no response or discrepant values were considered to be invalid data. Thus, 2,696 students (857 children and 1,839 adolescents) were studied. In relation to the initial number calculated (3,100), this study presented a data loss rate of 13%. Table 1 describes the data on the population studied.

**Table 1:** Description of explanatory and outcome variables among the students investigated and their families. Florianópolis, Santa Catarina, 2007

Variables	7-9 years old		10-14 years old	
	n	%	n	%
Gender				
Male	412	48.1	866	47.1
Female	445	51.9	973	52.9
Birth weight (g)				
< 3,000	242	28.2	490	26.6
3,000-3,999	552	64.4	1203	65.4
4,000	63	7.4	146	7.9
Birth weight according to gestational age				
SGA*	113	13.2	217	11.8
AGA*	594	69.3	1237	63.7
LGA*	80	9.3	215	11.7
Income quartiles (R$)/*per capita*				
1^st^	178	20.8	370	20.1
2^nd^	181	21.1	440	23.9
3^rd^	180	21.0	405	22.0
4^th^	181	21.1	387	21.0
Type of school				
Public	646	75.4	1373	74.7
Private	211	24.6	466	25.3
Parental nutritional status				
Obese mother	67	7.8	190	10.3
Obese father	83	9.7	189	10.3
Mother’s schooling level (years)				
< 8	180	21	466	25.3
8-11	375	43.8	794	43.2
12	273	31.9	540	29.4
Father’s schooling level (years)				
< 8	169	19.7	392	21.3
8-11	351	41.0	720	39.2
12	259	30.2	553	30.1

* SGA = small for gestational age; AGA = appropriate for gestational age; LGA = large for gestational age.

The LBW prevalences among the children and adolescents were respectively 8.2% and 7.8%. HBW was found in 7.4% and 7.9% of the children and adolescents, respectively. The prevalences of children and adolescents who were born SGA were 14.4% and 13.0%, respectively. Children and adolescents who were born LGA accounted for respectively 10.2% and 12.9%. 

The prevalence of overweight among the children was 31.5% and of obesity, 10.9%. Among the adolescents, the prevalence of overweight was 21.0% and of obesity, 6.0%. Table 2 shows the prevalences of overweight and obesity among the children according to gender and the variables investigated. In this table, it can seen that the prevalence of overweight among children was significantly higher in the following cases: boys born LGA (P = 0.02); children of both genders whose mothers were obese (P = 0.008 for boys and 0.001 for girls); and boys whose fathers were obese (P = 0.02). The prevalence of obesity was higher among girls whose mothers were obese (P < 0.001); and among boys whose fathers were obese (P = 0.05). The other variables investigated did not demonstrate any significant association with overweight or obesity in children (Pearson chi-square test).

**Table 2:** Prevalence of overweight and obesity according to explanatory variables, by gender, among schoolchildren aged 7-9 years. Florianópolis, Santa Catarina, 2007

Variables	Overweight (%)		Obesity (%)	
	Male	Female	Male	Female
Birth weight (g)				
< 3,000	27.3	27.2	12.6	5.4
3,000-3,999	34.3	31.2	15.1	8.3
4,000	41	26.3	13.6	10.5
Birth weight according to gestational age				
SGA	19.5	27.7	17.7	2.7
AGA	31.2	30.1	12.6	8.4
LGA	45.1^*^	41.4	17.5	13.8
Income quartiles (R$)/per capita				
1^st^	30	31.6	7.5	6.1
2^nd^	34.5	30.9	17.8	8.2
3^rd^	28.1	17.5	13.4	4.4
4^th^	32.9	34.4	17.5	7.8
Type of school				
Public	32.9	28.6	13.6	7.6
Private	34.8	33.3	16.5	6.8
Parental nutritional status				
Obese mother	57.1^†^	53.1^‡^	20.0	28.1^§^
Obese father	42.5^||^	41.8	15.0^¶^	13.9
Mother’s schooling level (years)				
< 8	37.8	25.5	15.5	6.6
8-11	32.9	32.1	13	8.5
12	31.8	29.7	16.3	6.5
Father’s schooling level (years)				
< 8	31.6	27.8	10.1	5.5
8-11	33.9	32.2	16.6	9.8
12	37.4	28.1	15.2	6.2

M = males

F = female

SGA = small for gestational age

AGA = appropriate for gestational age

LGA = large for gestational age

* P = 0.02

† P = 0.008

‡ P = 0.001

§ P < 0.001

|| P = 0.02

¶ P = 0.05

χ2 test

Table 3 shows the prevalences of overweight and obesity among the adolescents (10-14 years of age). The prevalence of overweight was higher among the following: boys with HBW (P = 0.01) and LGA (0.01); boys in the top monthly *per capita* income quartile (P = 0.01); boys whose mothers (P = 0.003) and fathers (P < 0.001) were obese; girls who attended public schools (P = 0.001); and girls whose mothers (P < 0.001) or fathers (P = 0.002) were obese. The prevalence of obesity was higher among the following: adolescents with HBW (P = 0.002); LGA (P = 0.001); and those whose mothers (P < 0.001) or fathers (P = 0.03) were obese (Pearson chi-square test).

**Table 3:** Prevalence of overweight and obesity according to explanatory variables, by gender, among schoolchildren aged 10-14 years. Florianópolis, Santa Catarina, 2007

Variables	Overweight (%)		Obesity (%)	
	Male	Female	Male	Female
Birth weight (g)				
< 3,000	21.8	13.5	7.4	1.7
3,000-3,999	26.8	16.3	8.8	3.1
4,000	37.3^*^	21.2	19.3^†^	4.2
Birth weight according to gestational age				
SGA	18.1	16.4	5.1	2.1
AGA	25.6	14.4	8.2	2.7
LGA	34.7^‡^	21.6	17.8^§^	4^||^
Income quartiles (R$)/per capita				
1^st^	18.5	17.2	7.3	1.5
2^nd^	26.6	17.2	10.7	3.5
3^rd^	25.9	16.6	10	3.7
4^th^	33.5^¶^	15.6	10	2.7
Type of school				
Public	25.4	17.3^**^	10.1	2.7
Private	31.4	11	8.5	2.7
Parental nutritional status				
Obese mother	38.7^††^	29.9^‡‡^	21.5^§§^	9.3^||||^
Obese father	41.0^¶¶^	27.6^***^	15.9	8.5^†††^
Mother’s schooling level (years)				
< 8	2.8	23.4	23.4	10.5
8-11	2.7	26.8	26.8	8.8
12	3.0	29.5	29.5	10.3
Father’s schooling level (years)				
< 8	2.4	22.2	22.2	8.4
8- 11	4.3	26.7	26.7	9.6
12	1.7	29.5	29.5	8.6

SGA = small for gestational age

AGA = appropriate for gestational age

LGA = large for gestational age

* P = 0.01

† P = 0.001

‡ P = 0.001

§ P = 0.001

|| P = 0.01

¶ P = 0.001

** P < 0.001

†† P = 0.003

‡‡ P < 0.001

§§ P < 0.001

|||| P = 0.002

¶¶ P < 0.001

*** P = 0.003

††† P = 0.03

These data show that a higher number of variables were associated with overweight and obesity among adolescents (12) than among children (6). Among children, the biological variables (birth weight for boys and parental BMI for both genders) were those that established a positive, proportional and significant association with the prevalences of overweight and obesity. Among adolescents, socioeconomic variables (kind of school and family income) were also associated in addition to the biological variables. The main variable of interest (birth weight) seemed to be associated only among males.

Table 4 shows the prevalence ratios in adjusted analysis on the outcomes of overweight and obesity among children and their associations with the exposure variables. No association between birth weight and overweight or obesity was found among the children in multivariate analysis. Thus, although birth weight according to gestational age was correlated with these outcomes in univariate analysis (Table 2), it ceased to be correlated after controlling for confounding. The father's and mother's BMI were probably the variables that were most strongly associated with the outcomes. 

**Table 4:** Prevalence ratio (PR) for overweight and obesity in adjusted analysis, according to categories of explanatory variables and according to gender, among children. Florianópolis, Santa Catarina, 2007

Variables	Overweight		Obesity	
	Male PR_aj _ (95% CI)*	Female PR_aj _ (95% CI)*	Male PR_aj _ (95% CI)*	Female PR_aj _ (95% CI)*
Birth weight (g)				
< 3,000	1.00	1.00	1.00	1.00
3,000-3,999	1.07 (0.96- 1.19)	0.99 (0.85-1.15)	1.10 (0.95-1.27)	0.99 (0.84-1.17)
4,000	1.02 (0.76- 1.36)	0.94 (0.75-1.17)	1.17 (0.88- 1.55)	0.90 (0.60-1.35)
Birth weight according to gestational age				
SGA	1.00	1.00	1.00	1.00
AGA	1.03 (0.78-1.37)	1.05 (0.97- 1.14)	0.91 (0.71-1.16)	1.06 (0.92-1.24)
LGA	1.15 (0.75-1.74)	1.01 (0.93- 1.09)	0.81 (0.54-1.22)	1.13 (0.68-1.87)

aj = adjusted

PR = prevalence ratio

SGA = small for gestational age

AGA = appropriate for gestational age

LGA = large for gestational age

* Analysis adjusted for socioeconomic variables (family income, type of school, i.e. public or private, and father's and mother's schooling levels) and biological variables (father's and mother's body mass index)

Table 5 shows the prevalence ratios for obesity among adolescents and their adjusted associations with birth weight. A significant association was found between birth weight and overweight among male adolescents, for those with HBW (PR = 1.14; 95% CI = 1.02-1.27; P = 0.03). However, it needs to be noted that this association observed between HBW and overweight among male adolescents was weak, because the prevalence of overweight among the male adolescents born with high birth weight was only 1.14 times greater than the prevalence of overweight found among adolescents born with appropriate weight. Hence, birth weight does not seem to be the principal factor that determines overweight in adolescence. Birth weight and birth weight according to gestational age were not significantly associated with obesity among adolescents. Again, other variables seem to be strongly associated with obesity, and parental BMI may have controlled this association. 

**Table 5:** Prevalence ratio (PR) for overweight and obesity in adjusted analysis, according to categories of explanatory variables and according to gender, among adolescents. Florianópolis, Santa Catarina, 2007

Variables	Overweight		Obesity	
	Male PR_aj _ (95% CI)*	Female PR_aj _ (95% CI)*	Male PR_aj _ (95% CI)*	Female PR_aj _ (95% CI)*
Birth weight (g)				
< 3,000	1.00	1.00	1.00	1.00
3,000-3,999	1.05 (0.92-1.20)	1.05 (0.93-1.19)	1.00 (0.94-1.06)	1.02 (0.98-1.05)
4,000	1.14 (1.02-1.27)	1.03 (0.92-1.15)	1.04 (0.98-1.10)	1.03 (0.97-1.08)
Birth weight according to gestational age				
SGA	1.00	1.00	1.00	1.00
AGA	1.01 (0.89-1.14)	0.93 (0.82-1.05)	0.98 (0.87- 1.11)	0.98 (0.94-1.01)
LGA	0.99 (0.87-1.12)	1.01 (0.90-1.14)	1.04 (0.87- 1.25)	0.99 (0.96-1.02)

aj = adjusted

PR = prevalence ratio

SGA = small for gestational age

AGA = appropriate for gestational age

LGA = large for gestational age

* Analysis adjusted for socioeconomic variables (family income, type of school, i.e. public or private, and father's and mother's schooling levels) and biological variables (father's and mother's body mass index).

## DISCUSSION

Investigating the influence of birth weight on overweight and obesity among children and adolescents is important, according to public health studies. This study in Florianópolis was justified by the city's high Human Development Index, which reached the fourth position in Brazil in 2000 and this index is continually increasing. Currently, Florianópolis is the third city in the country in terms of human development.^19^ ^,^ ^20^ Moreover, the prevalence of newborns who were LGA (10.2% among the children and 12.9% among the adolescents), was similar, in 2000, to that found in developed countries.^17^ ^,^ ^18^

The LBW prevalence found in this study (around 8%) is similar to that recorded in the Ministry of Health's Live Births Registry for the city of Florianópolis, which was 8.3% in 2005,^16^ and the same has been found in developing countries such as Argentina and Mexico.^35^ However, it must be pointed out that intrauterine growth restriction, which is an indication of poor nutrition during pregnancy, reached 27.4% in this study. 

The prevalences of overweight and obesity found in this survey (31.5% and 21% of the children and adolescents, respectively, were overweight and 10.9% and 6% were obese) were similar to those found in other studies on schoolchildren that used the same diagnostic criteria. For example, an assessment made by Dutra et al.^5^ on 810 adolescents aged 10 to 19 years in the city of Pelotas found that 19.3% of this population were overweight. Monteiro et al.,^3^ also in Pelotas, found that 20.5% were overweight and 7.7% were obese, among 1,076 schoolchildren aged 14 to 16 years. Among 2,936 schoolchildren aged 7 to 10 years assessed by de Assis et al.^14^ in Florianópolis in the year 2002, 10.6% were obese [BMI/age ≥ 95^th^ percentile of Must et al.^27^] Elevated proportions of overweight including obesity also were found among preschool children (28.8%) in Taubaté (state of São Paulo) and of obesity among children less than two years of age (10%) in Campinas (state of São Paulo).^36^ ^,^ ^37^ These findings may indicate that overweight and obese infants also tend to have overweight and obesity during childhood and adolescence.

Regarding the influence of birth weight on the prevalence of overweight in the sample investigated, a significant association was found in the univariate Poisson regression analysis for male adolescents who were born LGA. No association between birth weight and overweight was found among children of both genders, or among female adolescents. In an analysis adjusted for the parental socioeconomic and biological variables, the most significant association found was between HBW and overweight among male adolescents. Additionally, associations with obesity were found in univariate analysis for male adolescents who were born LGA and for those with HBW. However, after inclusion of parental socioeconomic variables and parental BMI in the multivariate analysis, the associations with obesity ceased to be significant.

Gilman et al.^4^ conducted a cross-sectional study on a cohort of 7,981 children and adolescents aged 9 to 14 years in the United States and found that the odds ratio for overweight [(BMI ≥ 85^th^ percentile and < 95] was associated with HBW [odds ratio (OR) = 1.2; 95% confidence interval (CI) = 1.1-1.4] in a multivariate model adjusted for socioeconomic, biological and behavioral variables. In Mexico, Moraes et al.^38^ conducted a cross-sectional study assessing 700 children and adolescents aged 5 to 13 years and found that birth weight between 2,890 and 3,110 g and birth weight ≥ 3,110 g were associated with overweight according to the cutoff points set by Cole et al.^15^ (OR = 2.85; 95% CI = 1.49-5.47; and OR = 7.03; 95% CI = 3.53-13.99, respectively), in a multivariate analysis. 

Monteiro et al.^3^ obtained similar results to those of the present study, in a cross-sectional investigation embedded in a cohort of children born in the city of Pelotas in 1982. Using the same diagnostic criteria for overweight and obesity as used in this study, they also found through a univariate analysis that there was an association between birth weight according to gestational age and obesity (i.e. being born LGA) among adolescents aged 14 to 16 years. However, the association did not remain significant after adjusting for family income and maternal BMI. 

The present study showed that being born with high birth weight is a factor associated with overweight among male adolescents. However, this association seemed to be weakened by the variables of obesity in the mother and obesity in the father, in the multivariate analysis. In childhood, this association between birth weight and overweight/obesity was not observed. This seems biologically plausible, since there are reports in the literature showing a strong association between these variables in adulthood.^39^ So, even though it was not so strong, the relationship between overweight in male adolescents and being born with high birth weight indicates that health services should promote nutritional monitoring among adolescents with a focus on lifestyle, in order to reduce the chances of overweight and obesity in adulthood. In addition, it is recommended that epidemiologists and other researchers should investigate whether adolescents with elevated birth weight were born from mothers who presented gestational obesity or gestational diabetes, because the origins of overweight may be found in intrauterine development.

In relation to birth weight and parental weight and height, it should be stressed that this information was reported by the children's legal guardians through a self-administered questionnaire. This method was chosen in order to make it easier to collect data and administer the data collection instrument. Araújo et al. conducted a validation study on the degree of agreement between reported birth weight information and birth weight measurements made immediately after birth, among eleven-year-old adolescents who were part of a cohort in Pelotas (Rio Grande do Sul) and found a high level of agreement regarding information on low birth weight (kappa = 0.73), with disagreements of the order of -20.0 g (standard deviation = 288.3).^40^ However, when stratifying birth weight information, the authors found that reported information tended to be overestimated in the case of LBW children and underestimated in the case of HBW children. They pointed out that the linear relationship between birth weight and BMI was more consistent when birth weight was measured immediately after birth and not so precise when birth weight was reported. Therefore, validation for birth weight measurements in a subsample is highly recommended, so as to compare the reported variable with data gathered by hospital registries or on health record cards.

In spite of these limitations, the present study had external validity, was probabilistic and had a complex sampling plan. Additionally, the researchers took care to train the anthropo-metry technicians, which resulted in reliable data. In addition to appropriate data gathering, care was taken in compiling the research data, which were fully entered and checked by a trained team, using software that enables data entry control. Therefore, the results from this study seem not to have been influenced by selection or measurement bias. 

## CONCLUSION

No significant association was found between high birth weight/being born large for gestational age and obesity after adjusting for the control variables, either among children or among adolescents. The same results were found for low birth weight and being born small for gestational age. Even though the association was not very strong, a relationship between high birth weight and overweight among male adolescents was observed.
